# Extraction optimization, purification and characterization of polysaccharides from the seed coat of black soybean

**DOI:** 10.1371/journal.pone.0190202

**Published:** 2017-12-21

**Authors:** Bao-yu Hu, Jun-cai Deng, Cai-qiong Yang, Yao Hu, Jing Zhang, Wen-yu Yang, Jiang Liu

**Affiliations:** 1 Key Laboratory of Crop Ecophysiology and Farming System in Southwest, Ministry of Agriculture, Chengdu, China; 2 Institute of Ecological Agriculture, Sichuan Agricultural University, Chengdu, China; College of Agricultural Sciences, UNITED STATES

## Abstract

In this study, the extraction of water-soluble polysaccharides from the seed coat of black soybean (BSCP) was investigated and optimized. A response surface methodology based on a Box-Behnken design (BBD) was used to optimize the extraction conditions as follows: extraction temperature, 100°C; ratio of water to material, 22.3 mL/g; and extraction time, 133.2 min. Under these conditions, the experimental yield of polysaccharides was 10.56%, which was consistent with the predictive yield. A novel galactomannan, BSCP-1, with a molecular weight of 7.55 × 10^5^ Da determined by high-performance gel permeation chromatography, was isolated from the black soybean seed coat. Through gas chromatography-mass spectrometry analysis, BSCP-1 was identified as a galactomannan consisting of galactose, mannose and rhamnose in a molar ratio of 6.01:3.56:1.00. Cytotoxicity against the human gastric carcinoma cancer cell line was also determined.

## Introduction

Black soybean seed is a well-known homologous plant food and medicine and is officially listed in the Chinese pharmacopoeia (2015 edition) [1]. This seed has historically been used to relieve kidney disease by increasing blood circulation and water passage, to counteract toxic effects, and for its antiaging effects, among other uses, in China, India, Japan, Korea and other Asian countries [2]. Sufficient studies have shown that black soybean seed is rich in polyphenolic substances, especially isoflavones [3] and anthocyanins, which are contained in most parts of the seed but especially in the seed coat [4]. Modern pharmacological studies have demonstrated that black soybean exhibits various bioactivities, including antioxidant and anti-inflammatory activities, reduction of DNA damage, and inhibition of low-density lipoprotein oxidation, which are primarily attributed to the anthocyanins with medium polarity [5]. Because of the various benefits of anthocyanins mentioned above, more research has focused on the alcohol-soluble component of black soybean seed. However, research on the high-polarity, water-soluble component has been relatively limited, especially regarding the structural elucidation of polysaccharides even though these compounds have proven to have health benefits for humans by lowering blood cholesterol, improving laxation and reducing the risk of diabetes, which was known before the 1990s [6]. Regarding the processing of black soybean seed, the soybean curd residue (namely, okara) is the dominant surplus material and is always regarded as waste [7].

Because of the attractive physicochemical properties of soybean polysaccharides, studies have sought to reveal their potential in biomaterial and biological applications, such as soybean polysaccharide films for food packaging [6, 8, 9]. At the same time, more research has also focused on the polysaccharides from black soybean seed. Recently, Jun Liu et al. isolated three water-soluble polysaccharide fractions, named BSPS-1, BSPS-2 and BSPS-3, from black soybean seed [10]. BSPS-1 was determined to be a linear (1→6)-α-D-glucan with a molecular weight of 1.95 × 105 Da, and BSPS-3 was found to be a type II arabinogalactan with a molecular weight of 1.88 × 105 Da. These two polysaccharides have been demonstrated to possess potential superoxide anion and DPPH radical scavenging activities [11].

Continuous rain during harvest can easily lead to damage to soybean plants due to field mold (FM), with *Fusarium moniliforme* confirmed as the main pathogen [12]. In our recent studies on FM in preharvest soybean seed, we found that mildew resistance was significantly correlated with the seed coat color and that soybean seeds with dark coats showed higher field mold resistance [13]. Although the obtained evidence confirmed that the antimycotic constituent primarily originated from the medium-polarity fraction [14], we believe that new functional constituents, especially polysaccharides, are contained in the wasted high-polarity, water-soluble fraction of the black soybean seed coat. In the present study, a three-level, three-factor Box-Behnken design was combined with response surface methodology (RSM) to optimize the extraction conditions for obtaining the maximum yield of BSCPs. In addition, preliminary structural characterization was carried out, and the in vitro anti-tumor activities of the BSCPs were investigated.

## Materials and methods

### Materials and chemicals

The black soybean cultivar “C103” (*Glycine max* L. Merr.), a conventional high-resistance cultivar in southwestern China, was evaluated in the experiments [15]. The soybeans were grown in an experimental field of Sichuan Agricultural University at Ya’an in China (103°00′E, 30°08′N). The soybeans were sown on 24 June 2014, and the seeds of C103 were harvested on 5 November 2014 at full maturity (R8) [16] and dried naturally. Standard compounds of rhamnose (RHA), arabinose (ARA), fucose (FUC), xylose (XYL), mannose (MAN), glucose (GLU), galactose (GAL), fructose (FRU) and deuterium oxide (D_2_O) were all purchased from Sigma Chemical Co. (St. Louis, MO, USA). Other reagents were of analytical grade.

### Extraction of polysaccharides and determination of the associated yields

First, 2.0 kg of seed coat was mechanically exfoliated from natural dried black soybean seeds. All of the black soybean seed coat was extracted three times with 20 L of methanol under reflux for 3 h. Then, the dried filtered residues (0.5 g) without methanol were continuously extracted twice with distilled water using different extraction times, temperatures and ratios of water to raw material. Finally, distilled water was added to bring the filter liquor from the extraction to 100 mL to prepare the sample liquor. The total polysaccharide yield from the soybean seed coat was determined using the phenol-sulfuric acid method. The yield results were expressed as glucose equivalents [14].

### Extraction optimization using RSM

The effects of the extraction time, temperature and ratio of water to raw material were analyzed using the RSM. After three single-factor experiments, the preliminary ranges of the extraction variables were determined. The independent variables were the extraction time (90, 120 and 150 min), extraction temperature (90, 95 and 100°C) and the ratio of water to raw material (15:1, 20:1 and 25:1). Then, a Box-Behnken factorial design (BBD) was adopted in which a 15-run BBD with three variables and three levels, including five replicates at the center point, was used to optimize the extraction conditions [17]. The symbols and coded factor levels for these variables are given in Table 1. The experimental design and regression analysis were conducted using Design-Expert software (version 8.0, Stat-Ease Inc., Minneapolis, USA). The interrelationships of the variables were based on a second-order polynomial model [10].

**Table 1:** Coded levels and real values for the BBD and the extraction yield results.

Standard order	Coded levels (real values)			Extraction yield (%)	
	A: Extraction temperature (°C)	B: Ratio of water to material (mL/g)	C: Extraction time (min)	Observed	Predicted
1	-1(90)	-1(15)	0(120)	9.23	9.16
2	-1(90)	1(25)	0(120)	9.88	9.69
3	0(95)	-1(15)	0(120)	9.55	9.74
4	0(95)	1(25)	0(120)	10.27	10.34
5	-1(90)	0(20)	-1(90)	9.02	10.11
6	-1(90)	0(20)	1(150)	9.98	10.15
7	1(100)	0(20)	-1(90)	10.32	10.15
8	1(100)	0(20)	1(150)	10.43	10.34
9	0(95)	-1(15)	-1(90)	9.12	9.10
10	0(95)	-1(15)	1(150)	9.40	9.30
11	0(95)	1(25)	-1(90)	10.25	10.27
12	0(95)	1(25)	1(150)	10.26	10.27
13	0(95)	0(20)	0(120)	10.12	10.12
14	0(95)	0(20)	0(120)	10.13	10.12
15	0(95)	0(20)	0(120)	10.12	10.12

### Purification of the crude BSCP fraction

The dried filtered residues (1.5 kg) resulting from methanol extraction were extracted with boiling water three times (2 h each) and then filtered. The filtrate was concentrated and dialyzed against running water with a tubular membrane (MWCO: 3500 Da, Jinhui Biotechnology Company, Shanghai, China) for 2 days. The dialysis products were dried under a vacuum, and the crude polysaccharide fraction was obtained [18]. Part (1.0 g) of the crude polysaccharide fraction was dissolved in deionized water and loaded onto a fast flow DEAE-Sepharose CL-6B column (32×450 mm), eluting successively with 0.03 M, 0.06 M, 0.18 M, 0.36 M, 0.72 M and 1.5 M NaCl (500 mL each). The polysaccharide content was determined using the phenol-sulfuric acid method.

### Determination of the purity and molecular weight

High-performance gel permeation chromatography (HPGPC) was used to detect the purity and molecular weights of the BSCPs. The analyses were performed on an LC-6AD system (Shimadzu, Japan) equipped with an RID-10A refractive index detector (Shimadzu, Japan) and an Agilent ZORBAX GF-250 size exclusion analytical column (4.6×250 mm, 4 μm), which was eluted with ultrapure water at a flow rate of 0.2 mL/min; the injection volume was 20 μL. The molecular weights of the polysaccharide samples were calculated from the calibration curve generated using a series of dextran standards (T-500, T-100, T-70, T-40 and T-10) with definite molecular masses ranging from 10 to 500 kDa [18].

### Analysis of the monosaccharide composition

The monosaccharide composition was determined by gas chromatography-mass spectrometry (GC-MS) after derivatization to form aldononitrile acetates [19]. Briefly, the BSCPs (10 mg) were hydrolyzed with 2 mL of 2 M trifluoroacetic acid at 100°C for 6 h. The vacuum-dried hydrolysate was prepared by adding 0.5 mL of pyridine and 10 mg of hydroxylamine hydrochloride with heating at 90°C for 30 min. After cooling to room temperature, 0.8 mL of acetic anhydride was added to the above mixture, and heating at 90°C was continued for 30 min. The entire mixture was vacuum-dried and then re-dissolved in 5 mL of chloroform. The samples were then centrifuged at 11,000 g and 4°C for 5 min, and approximately 1.5 mL of the supernatant was filtered through a 0.22-μm syringe filter and stored at -20°C before injection into the GC-MS system.

The aldononitrile acetate derivatives of the standard sugars (RHA, ARA, FUC, XYL, MAN, GLU, GAL and FRU) were prepared in the same way. Samples were analyzed on a GC-MS instrument with a QP2010 system (Shimadzu, Japan). Separation was performed on a capillary column (Rtx-5Ms, 30 m×0.25 mm×0.25 μm). The injection port temperature was set to 280°C, and helium was used as the carrier gas at a flow of 1.0 mL·min^-1^; the injection volume was 1.0 μL. The ionization potential of the mass-selective detector was 70 eV, and the scan range was 35–550 amu. The oven temperature was increased from 130 to 230°C at a rate of 3.0°C·min^-1^, followed by an isothermal hold for 2 min. All samples were analyzed in triplicate. The compounds were identified by comparing the retention times and mass spectra of the samples with those of the standards.

### In vitro cytotoxicity assays

BSCP-1 was tested for its cytotoxic activity against the HGC27 human gastric carcinoma cancer cell line using the CellTiter-Glo^™^ luminescent cell viability assay (Promega) [20]. The HGC27 cell line was cultured in RPMI-1640 medium supplemented with 10% fetal bovine serum (FBS). The cells were maintained at 37°C in a humidified environment containing 5% CO_2_. Cell viability was determined using the CellTiter-Glo^™^ assay. Briefly, the cancer cells were seeded onto 384-well plates at an initial density of 2000 cells/well in 40 μL of medium. Then, the cells were treated with the compounds at varying concentrations. The dose-effect curve of doxorubicin cytotoxicity against the BEL-7402 cells was used as a positive control, and 0.5% DMSO was used as a negative control. After incubation for 72 h, 30 μL/well of CellTiter-Glo^™^ reagent was added, and the relative light unit (RLU) values were read on a PHERAstar^plus^ microplate reader (BMG Labtech, Cary, NC) after incubation for 10 min at room temperature: cell growth inhibition % = 100% × [1-RLU_treatment_/RLU_negative control_]. The IC_50_ values were calculated from the curves generated by plotting cell growth inhibition versus the test concentrations using GraphPad Prism 6.0 software [21].

## Results and discussion

### Optimization of extraction conditions by single-factor experiments

**Fig 1:**
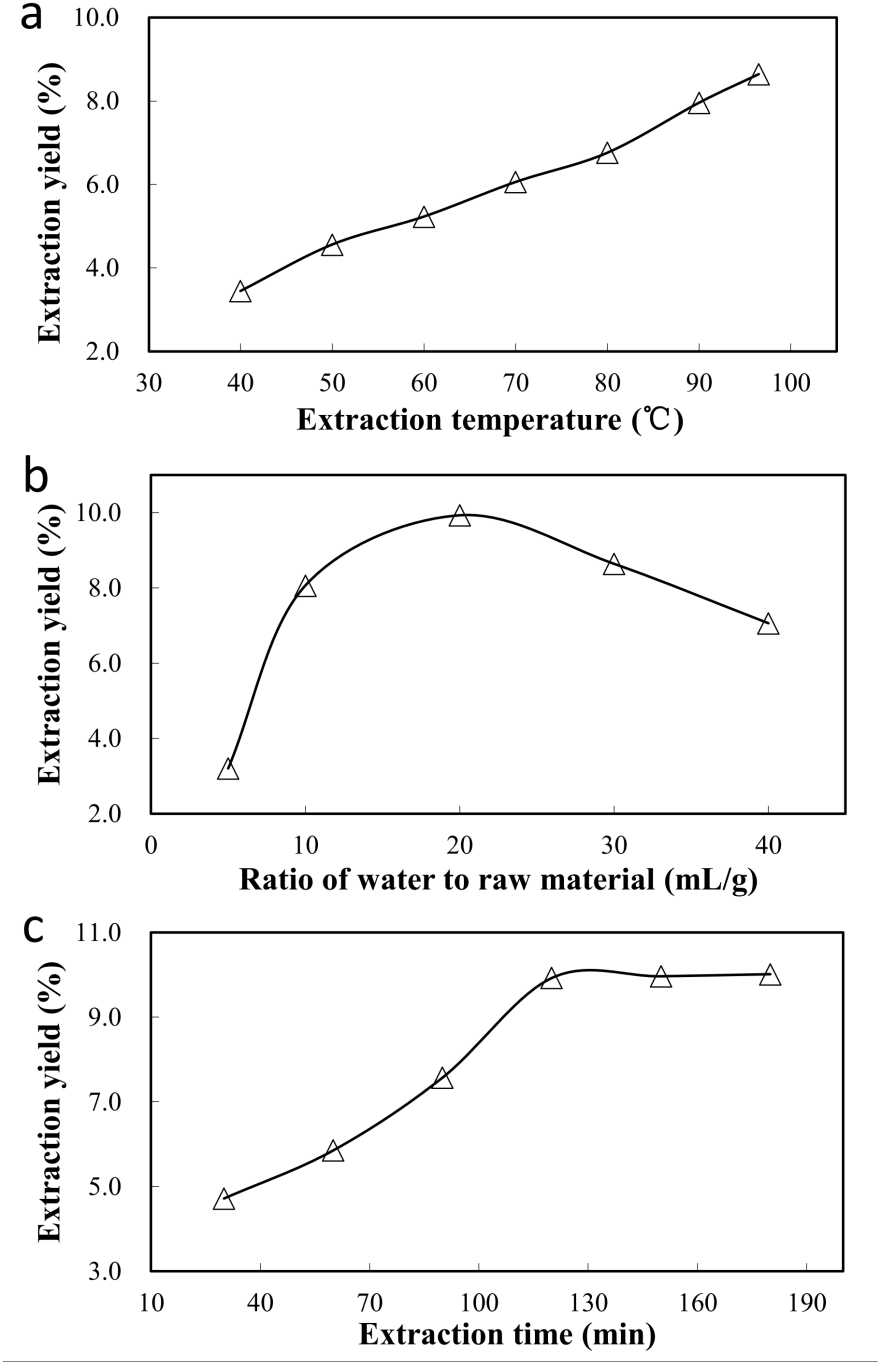
Effects of the extraction temperature (a), ratio of water to raw material (b) and extraction time (c) on the yield of crude BSCPs.

The effects of the extraction parameters on the yield of crude BSCPs are shown in Fig 1. The effect of the extraction temperature is shown in Fig 1a. When the extraction time and ratio of water to raw material were fixed at 120 min and 30 mL/g, respectively, the extraction yield of BSCPs increased rapidly from 3.78% to 8.64% as the extraction temperature increased from 40 to 100°C, indicating that boiling water facilitated the release of BSCPs. Similarly, the effect of the ratio of water to raw material on the yield of crude BSCPs is shown in Fig 1b. When the extraction time and temperature were fixed at 120 min and 100°C, respectively, the extraction yield of BSCPs increased rapidly as the ratio of water to raw material increased from 5 to 20 mL/g, peaked at 20 mL/g, and decreased when the ratio exceeded 20 mL/g. Additionally, when the extraction temperature and the ratio of water to raw material were fixed at 100°C and 20 mL/g, respectively, the extraction yield increased as the extraction time increased from 30 to 120 min, peaked at 120 min, and did not significantly increase further when the extraction time exceeded 120 min (see Fig 1c).

### Optimization of the extraction yield by RSM

The appropriate ranges of the extraction temperature (90, 95 and 100°C), ratio of water to material (15, 20 and 25 mL/g) and extraction time (90, 120 and 150 min) for the crude BSCP extraction were determined in the single-factor experiments. From these results, the extraction parameters were further investigated for the optimum conditions using a BBD [22]. The experimental conditions and the observed and predicted BSCP yields according to the BBD are listed in Table 1. The BSCP yield ranged from 9.02% to 10.43%. The predicted and observed values were highly similar under the same extraction conditions, which reflected the accuracy and applicability of a BBD for the optimization of the BSCP extraction yield. Multiple regression analysis was applied to the experimental data, and the response variables and test variables were correlated according to the following second-order polynomial equation:

$$\text{Y}=10.12+0.31\text{A}+0.28\text{B}+0.31\text{C}+0.018\text{AB}-0.21\text{AC}+0.21\text{BC}+0.038{\text{A}}^{2}-0.42{\text{B}}^{2}-0.22{\text{C}}^{2},$$

where Y is the yield of BSCPs (%) calculated from the regression model, and A, B and C are the coded variables of the extraction temperature, ratio of water to material and extraction time, respectively.

**Fig 2:**
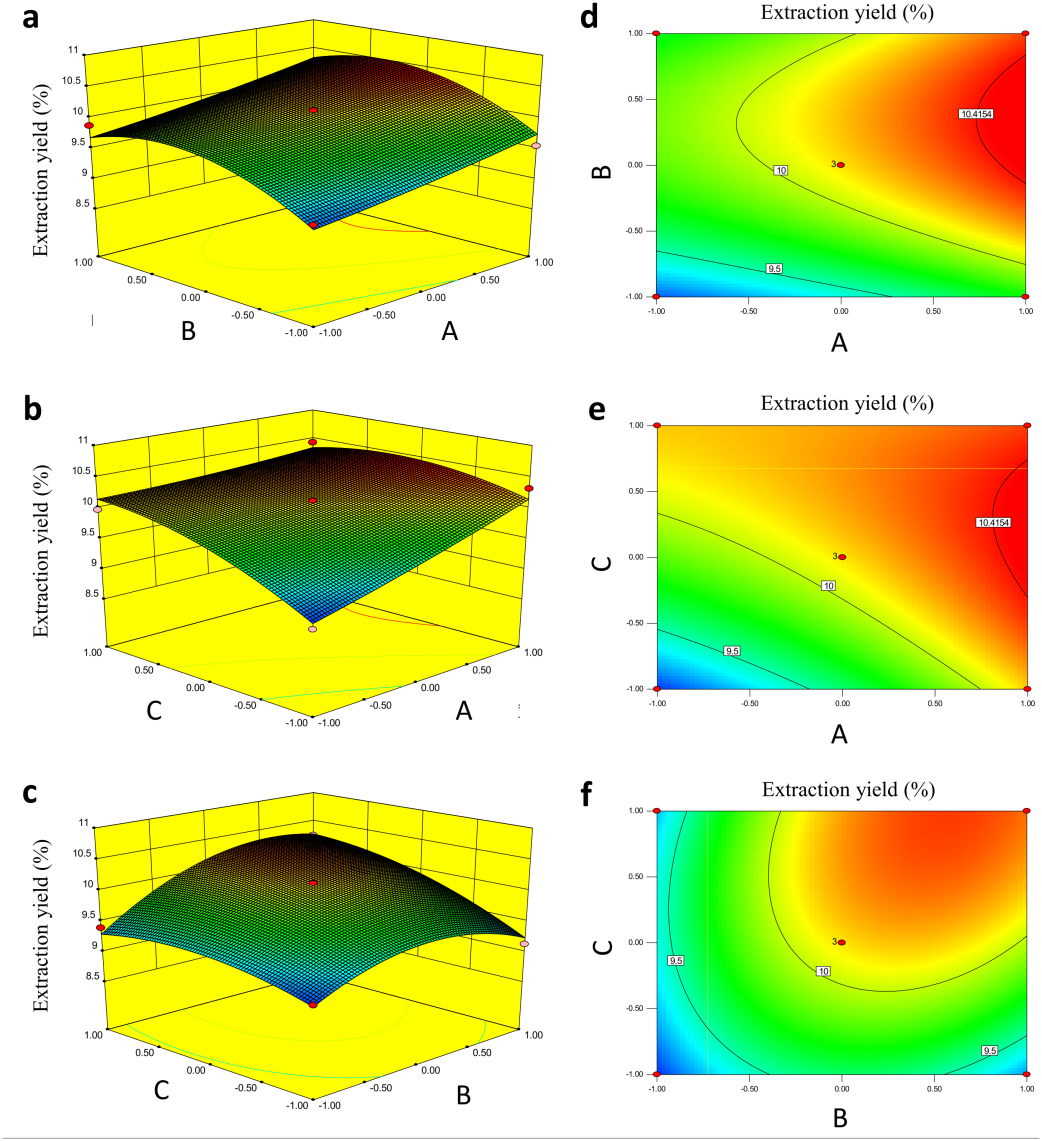
Three-dimensional response surfaces (a, b and c) and contour graphs (d, e and f) for the interactive effects of the extraction temperature (A), ratio of water to material (B) and extraction time (C) on the BSCP yield.

An analysis of variance (ANOVA) of the response surface quadratic model was conducted. As shown in Table 2, the high F value (F = 10.18) and low P value (*P* < 0.01) of the model indicate that the polynomial model was highly statistically significant. The high determination coefficient (*R*^2^ = 0.9482) indicates that 94.82% of the variability in the response could be explained by the model, demonstrating that the model equation had a high-quality fit and good precision and reliability [23]. Response surfaces were plotted using Design-Expert software to explain the interactions among the variables and to determine the optimal level of each variable for the maximum response. The three-dimensional response surfaces and two-dimensional contours are shown in Fig 2. Response surface analysis was performed using Design-Expert software to determine the following optimal extraction conditions: extraction temperature, 100°C; ratio of water to material, 22.3 mL/g; and extraction time, 133.2 min. The maximum predicted yield of BSCPs was 10.55%. To validate the model equations, a verification experiment under these conditions was conducted, and the experimental yield of BSCPs was 10.56 ± 0.0005% (mean±RSD, n = 3), which was highly consistent with the predicted value. The above result demonstrated that the regression model was accurate and adequate for the prediction of the BSCP extraction yield.

**Table 2:** ANOVA of the response surface quadratic model for the BSCP yield.

Source	Sum of square	Degree of freedom	Mean square	F value	P Value Prob. > F
A	0.76	1	0.76	20.86	0.0060**
B	0.62	1	0.62	17.14	0.0090**
C	0.76	1	0.76	21.03	0.0059**
AB	1.23	1	1.23	0.034	0.8614
AC	0.18	1	0.18	4.98	0.0760
BC	0.18	1	0.18	4.86	0.0785
A^2^	5.19	1	5.19	0.14	0.7207
B^2^	0.67	1	0.67	18.39	0.0078**
C^2^	0.18	1	0.18	4.93	0.0771
Model	3.32	9	0.37	10.18	0.0100
Residual	0.18	5	0.036		
Lack of fit	0.18	3	0.060		
Pure error	0.00	2	0.000		
Total	3.50	14			

** Indicates significance (significance level 0.01).

### Purification and molecular weight determination

**Fig 3:**
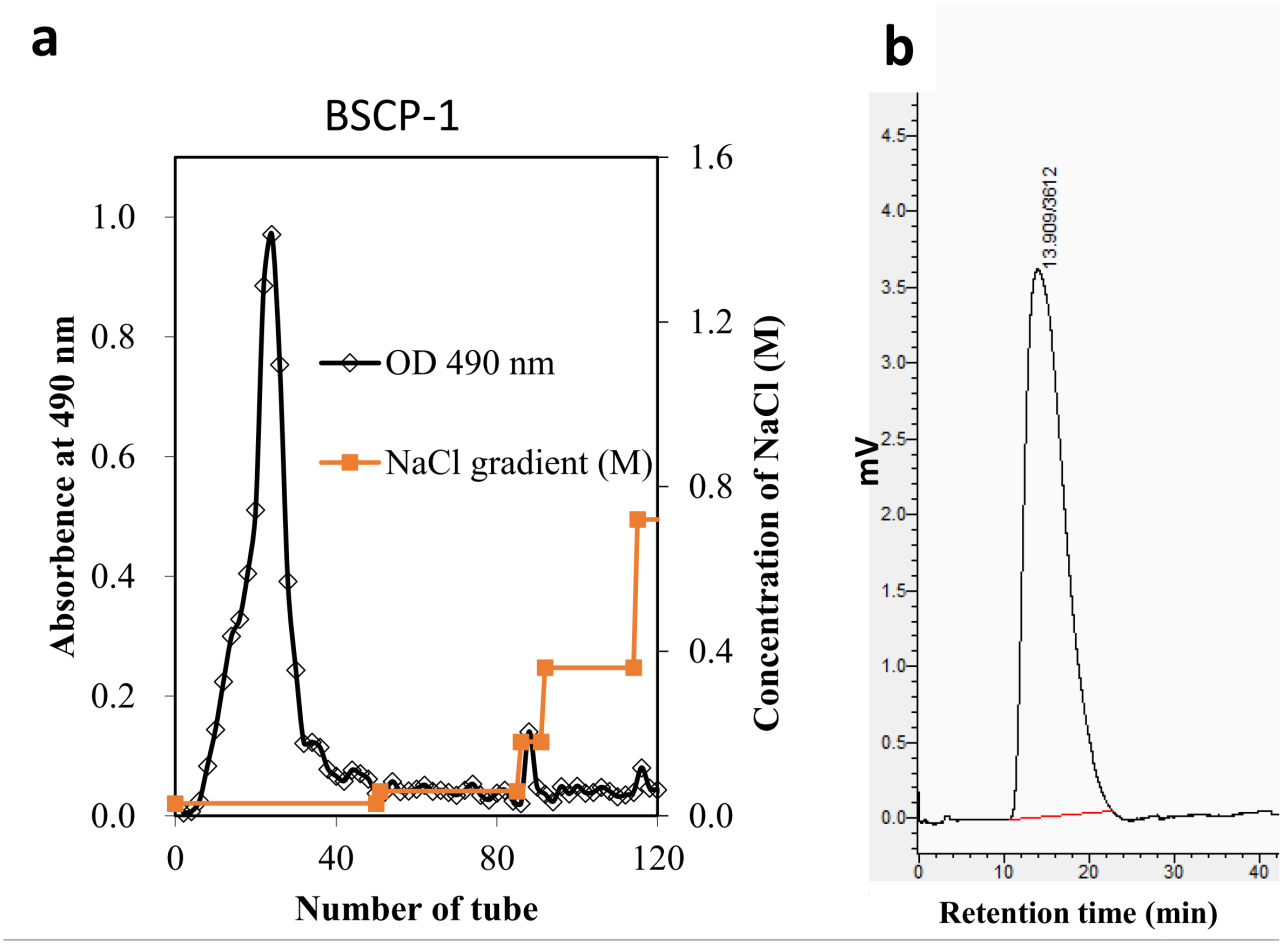
Elution profile of the crude BSCPs on a DEAE-Sepharose CL-6B chromatography column, eluting with an NaCl gradient at a flow rate of 2.5 mL/min (a); HPGPC result for BSCP-1 (b).

The crude BSCP fraction was separated on a fast flow DEAE-Sepharose CL-6B column, eluting successively with an NaCl gradient. From the elution profile of crude BSCPs on the DEAE-Sepharose CL-6B column (Fig 3a), main independent fraction peaks were collected at the elution of water, named BSCP-1. The homogeneous polysaccharide was further analyzed by HPGPC to detect the purity and molecular weight. As shown in Fig 3b, the BSCP-1 peak showed good symmetry, which indicated good homogeneity. Via calculation with the regression equation, lgMw = -1.032x+10.08 (*R*^2^ = 0.982), the molecular weight of BSCP-1 was determined to be 7.55 × 10^5^ Da.

### Monosaccharide composition analysis

**Fig 4:**
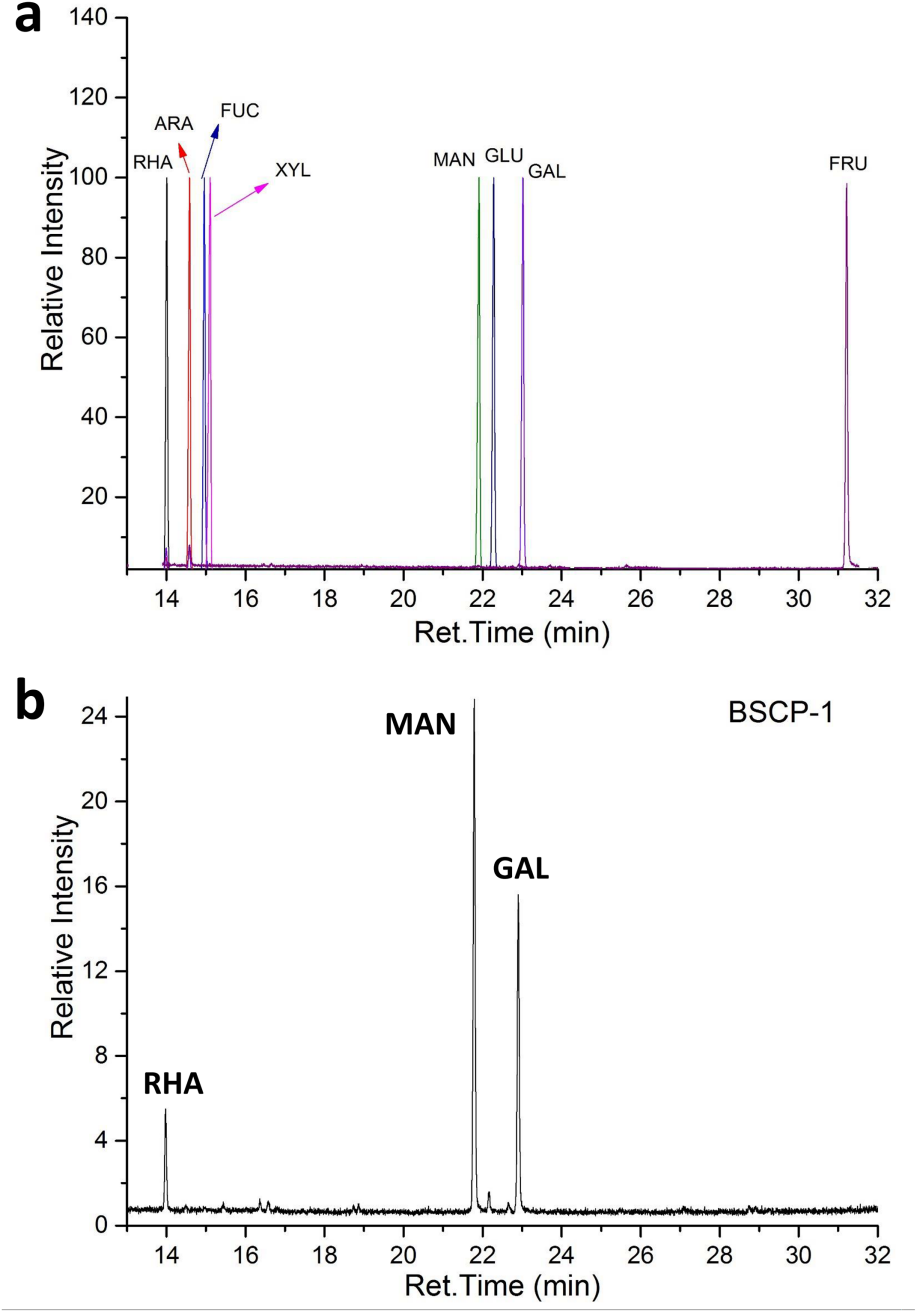
GC-MS chromatogram of the aldononitrile acetate derivatives of the standard monosaccharides (a) and the hydrolysate of BSCP-1 (b).

The aldononitrile acetate derivatives of the monosaccharide standards (Fig 4a) and hydrolyzed BSCP-1 were analyzed by GC-MS. As shown in Fig 4b, purified BSCP-1 was composed of rhamnose, mannose and galactose in a molar ratio of 1.00:6.01:3.56. In previous studies, several polysaccharides were isolated from black soybean seed, and the typical polysaccharide, named PSBS, was composed of 89% glucose, 6% mannose and 5% galactose [24]. BSPS-1 and BSPS-2 were predominantly composed of glucose and galactose, and BSPS-3 was predominantly composed of galactose and arabinose [7]. These data indicated that mannose and galactose were the predominant monosaccharides in the new polysaccharide BSCP-1.

### Cytotoxic activity against HGC27 cells

In the past five years, several polysaccharides composed of galactose, rhamnose and mannose have been reported to have potent anti-proliferation and anti-metastatic activities and could be developed as anti-tumor agents for human gastric [21], bladder [25] and lung [26] cancer therapy. In this study, the isolated BSCP-1 was tested for its in vitro cytotoxicity against the HGC27 human gastric carcinoma cancer cell line using the Cell-Titer-Glo^™^ luminescent cell viability assay. However, BSCP-1 showed no cytotoxic effects (IC_50_ >0.15 mg/ml) against the HGC27 cell line.

## Conclusion

In this paper, the extraction of BSCPs was optimized. The yield of BSCPs obtained under the optimal extraction conditions (extraction temperature, 100°C; ratio of water to material, 22.3 mL/g; and extraction time, 133.2 min) was 10.55%. The crude BSCP fraction was separated by DEAE-Sepharose column chromatography, and BSCP-1, containing pectic-type polysaccharide with a molecular weight of 7.55 × 10^5^ Da, was isolated from the black soybean seed coat. The detailed composition of BSCP-1 was identified through GC-MS analysis as a galactomannan consisting of galactose, mannose and rhamnose in a molar ratio of 6.01:3.56:1.00. Our results also indicated that BSCP-1 exhibited lower anti-tumor activities than other analogues. Further investigations must be performed to evaluate other possible biological activities of this galactomannan-rich polysaccharide to determine possible applications of black soybean seed coat.

## References

[1] China TSPCoPsRo. Pharmacopeia of the People’s Republic of China. Beijing: China Medical Science Press; 2015 344 p.

[2] Xu B, Chang SKC. Antioxidant Capacity of Seed Coat, Dehulled Bean, and Whole Black Soybeans in Relation to Their Distributions of Total Phenolics, Phenolic Acids, Anthocyanins, and Isoflavones. J Agric Food Chem. 2008;56(18):8365–73. doi: 10.1021/jf801196d 18729453 10.1021/jf801196d

[3] Liu J, Yang C-Q, Lou Y, Wu H-J, Deng J-C, Yang F, et al Partial improvements in the flavor quality of soybean seeds using intercropping systems with appropriate shading. Food chemistry. 2016;207:107–14. doi: 10.1016/j.foodchem.2016.03.059 27080886 10.1016/j.foodchem.2016.03.059

[4] Lee JH, Kang NS, Shin S-O, Shin S-H, Lim S-G, Suh D-Y, et al Characterisation of anthocyanins in the black soybean (Glycine max L.) by HPLC-DAD-ESI/MS analysis. Food Chem. 2008;112(1):226–31. doi: 10.1016/j.foodchem.2008.05.056

[5] Wu H-j, Deng J-c, Yang C-q, Zhang J, Zhang Q, Wang X-c, et al Metabolite profiling of isoflavones and anthocyanins in black soybean [Glycine max (L.) Merr.] seeds by HPLC-MS and geographical differentiation analysis in Southwest China. Analytical Methods. 2017;9(5):792–802. doi: 10.1039/C6AY02970A

[6] Salarbashi D, Tajik S, Shojaee-Aliabadi S, Ghasemlou M, Moayyed H, Khaksar R, et al Development of new active packaging film made from a soluble soybean polysaccharide incorporated Zataria multiflora Boiss and Mentha pulegium essential oils. Food chemistry. 2014;146:614–22. https://doi.org/10.1016/j.foodchem.2013.09.014. 24176389 10.1016/j.foodchem.2013.09.014

[7] Jia X, Chen M, Wan J-B, Su H, He C. Review on the extraction, characterization and application of soybean polysaccharide. RSC Advances. 2015;5(90):73525–34. doi: 10.1039/C5RA12421B

[8] Tajik S, Maghsoudlou Y, Khodaiyan F, Jafari SM, Ghasemlou M, Aalami M. Soluble soybean polysaccharide: A new carbohydrate to make a biodegradable film for sustainable green packaging. Carbohydrate polymers. 2013;97(2):817–24. https://doi.org/10.1016/j.carbpol.2013.05.037. 23911520 10.1016/j.carbpol.2013.05.037

[9] Salarbashi D, Tajik S, Ghasemlou M, Shojaee-Aliabadi S, Shahidi Noghabi M, Khaksar R. Characterization of soluble soybean polysaccharide film incorporated essential oil intended for food packaging. Carbohydrate polymers. 2013;98(1):1127–36. https://doi.org/10.1016/j.carbpol.2013.07.031. 23987454 10.1016/j.carbpol.2013.07.031

[10] Liu J, Wen X-y, Zhang X-q, Pu H-m, Kan J, Jin C-h. Extraction, characterization and in vitro antioxidant activity of polysaccharides from black soybean. International journal of biological macromolecules. 2015;72:1182–90. http://doi.org/10.1016/j.ijbiomac.2014.08.058. 25256548 10.1016/j.ijbiomac.2014.08.058

[11] Liu J, Wen X-y, Kan J, Jin C-h. Structural Characterization of Two Water-Soluble Polysaccharides from Black Soybean (Glycine max (L.) Merr.). Journal of Agricultural and Food Chemistry. 2015;63(1):225–34. doi: 10.1021/jf505172m 25494923 10.1021/jf505172m

[12] Liu J, Deng J-c, Yang C-q, Huang N, Chang X-l, Zhang J, et al Fungal Diversity in Field Mold-Damaged Soybean Fruits and Pathogenicity Identification Based on High-Throughput rDNA Sequencing. Frontiers in Microbiology. 2017;8:779 doi: 10.3389/fmicb.2017.00779 28515718 10.3389/fmicb.2017.00779 PMC5413577

[13] Liu J, Deng J-c, Yang C-q, Huang N, Chang X-l, Zhang J, et al Fungal Diversity in Field Mold-Damaged Soybean Fruits and Pathogenicity Identification Based on High-Throughput rDNA Sequencing. Frontiers in Microbiology. 2017;8(779). doi: 10.3389/fmicb.2017.00779 28515718 10.3389/fmicb.2017.00779 PMC5413577

[14] Liu J, Deng J, Zhang K, Wu H, Yang C, Zhang X, et al Pod Mildew on Soybeans Can Mitigate the Damage to the Seed Arising from Field Mold at Harvest Time. Journal of Agricultural and Food Chemistry. 2016;64(48):9135–42. doi: 10.1021/acs.jafc.6b03561 27933997 10.1021/acs.jafc.6b03561

[15] Liu J, Hu B, Liu W, Qin W, Wu H, Zhang J, et al Metabolomic tool to identify soybean [Glycine max (L.) Merrill] germplasms with a high level of shade tolerance at the seedling stage. Sci Rep. 2017;7:42478 doi: 10.1038/srep42478 . 28211897 10.1038/srep42478 PMC5304147

[16] Fehr WR, Caviness CE, Burmood DT, Pennington JS. Stage of Development Descriptions for Soybeans, Glycine Max (L.) Merrill1. Crop Science. 1971;11(6):929–31.

[17] Zou Y, Chen X, Yang W, Liu S. Response surface methodology for optimization of the ultrasonic extraction of polysaccharides from Codonopsis pilosula Nannf.var.modesta L.T.Shen. Carbohydrate Polymers. 2011;84(1):503–8. http://doi.org/10.1016/j.carbpol.2010.12.013.

[18] He Z, Liang F, Zhang Y, Pan Y. Water-soluble polysaccharides from finger citron fruits (Citrus medica L. var. sarcodactylis). Carbohydrate Research. 2014;388:100–4. http://doi.org/10.1016/j.carres.2013.12.020. 24632217 10.1016/j.carres.2013.12.020

[19] Zhang W, He H, Zhang X. Determination of neutral sugars in soil by capillary gas chromatography after derivatization to aldononitrile acetates. Soil Biology and Biochemistry. 2007;39(10):2665–9. http://dx.doi.org/10.1016/j.soilbio.2007.04.003.

[20] Wu S-B, Bao Q-Y, Wang W-X, Zhao Y, Xia G, Zhao Z, et al Cytotoxic Triterpenoids and Steroids from the Bark of Melia azedarach. Planta Med. 2011;77(09):922–8. Epub 17.01.2011. doi: 10.1055/s-0030-1250673 21243584 10.1055/s-0030-1250673

[21] Li C, Tian Z-N, Cai J-P, Chen K-X, Zhang B, Feng M-Y, et al Panax ginseng polysaccharide induces apoptosis by targeting Twist/AKR1C2/NF-1 pathway in human gastric cancer. Carbohydrate polymers. 2014;102:103–9. http://doi.org/10.1016/j.carbpol.2013.11.016. 24507261 10.1016/j.carbpol.2013.11.016

[22] Zhao Z-Y, Zhang Q, Li Y-F, Dong L-L, Liu S-L. Optimization of ultrasound extraction of Alisma orientalis polysaccharides by response surface methodology and their antioxidant activities. Carbohydrate polymers. 2015;119:101–9. https://doi.org/10.1016/j.carbpol.2014.11.052. 25563949 10.1016/j.carbpol.2014.11.052

[23] Han Y-L, Gao J, Yin Y-Y, Jin Z-Y, Xu X-M, Chen H-Q. Extraction optimization by response surface methodology of mucilage polysaccharide from the peel of Opuntia dillenii haw. fruits and their physicochemical properties. Carbohydrate polymers. 2016;151:381–91. https://doi.org/10.1016/j.carbpol.2016.05.085. 27474580 10.1016/j.carbpol.2016.05.085

[24] Liao HF. Isolation and characterization of an active compound from black soybean (Glycine max (L.) Merr.) and its effect on proliferation and differentiation of human leukemic U937 cells. Anticancer Drugs. 2001;12:841–6. 11707652 10.1097/00001813-200111000-00008

[25] Li C, Cai J, Geng J, Li Y, Wang Z, Li R. Purification, characterization and anticancer activity of a polysaccharide from Panax ginseng. International journal of biological macromolecules. 2012;51(5):968–73. https://doi.org/10.1016/j.ijbiomac.2012.06.031. 22750577 10.1016/j.ijbiomac.2012.06.031

[26] Wang Y, Huang M, Sun R, Pan L. Extraction, characterization of a Ginseng fruits polysaccharide and its immune modulating activities in rats with Lewis lung carcinoma. Carbohydrate polymers. 2015;127:215–21. https://doi.org/10.1016/j.carbpol.2015.03.070. 25965477 10.1016/j.carbpol.2015.03.070

